# WST-assay data reveal a pH dependence of the mitochondrial succinate reductase in osteoblast-like cells

**DOI:** 10.1016/j.dib.2017.04.037

**Published:** 2017-04-28

**Authors:** Anne-Marie Galow, Jan Gimsa

**Affiliations:** Chair for Biophysics, University of Rostock, Gertrudenstr. 11a, 18057 Rostock, Germany

**Keywords:** MC3T3 cell line, Proliferation, Enzyme-based assay, pHdependent readout, Alkaline medium

## Abstract

The data presented in this article are related to the research article entitled “Increased osteoblast viability at alkaline pH in vitro provides a new perspective on bone regeneration” (doi: 10.1016/j.bbrep.2017.02.001; (Galow et al., 2017) [1]). The water soluble tetrazolium (WST) proliferation assay detects the metabolic activity of the respiratory chain of cultured cells. The assay is based on changes in the light absorbance resulting from the metabolism of WST-1 into formazane by mitochondrial succinate reductase. We present data of three different tests that were carried out to check whether WST assay readouts are pH-dependent. In a first test, a possible pH effect on the photometric measurements, for example by shifting the absorbance spectrum of the pH indicator of the cell culture medium, was excluded. Because the second test revealed a significant pH-dependence of the activity of the mitochondrial succinate reductase, a third long-term test was conducted to analyze possible changes of the pH dependence over time. The higher absorbance per one million cells at alkaline pH, which was approximately four-fold at pH 8.4 compared to the pH-7.4 reference on day one decayed gradually, with the pH-differences equilibrating over six days.

**Specifications Table**

**Table:** 

Subject area	*Cell biology*
More specific subject area	*Bone cell biology, metabolism*
Type of data	*Table in MS Excel file, graph*
How data was acquired	*Microscope, photometer*
Data format	*Raw, analyzed*
Experimental factors	*Cells were cultured in α-MEM at pH 7.2, 7.4, 7.8, or 8.4 for one hour and up to 6 days*
Experimental features	*Cell culture, photometric measurements, WST-1 assay*
Data source location	*Does not apply*
Data accessibility	*The data are included with this article*

**Value of the data**

• Our data revealed pH limitations of enzyme-based proliferation assays, such as the WST assay.
• Our data suggest that cellular succinate reductase is more highly efficient at alkaline pH.
• Our data suggest pH corrections are required for the comparison of enzyme-based proliferation assays.
• Our MC3T3 osteoblast-like cell line data can be compared with data on other cell lines or primary cells under different pH conditions.
• Our data can be used to design further experiments on the cell-type-specific pH-dependence of cellular metabolism.

## 1 Data

**Fig. 1:**
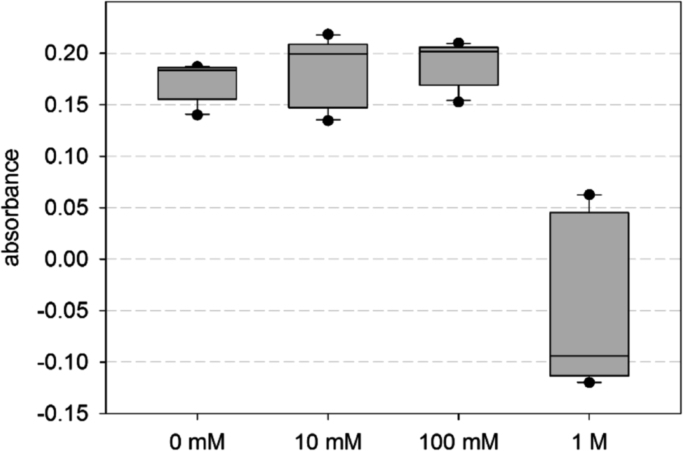
Absorbance at 450 nm, dependent on the NaOH concentration of the 10 µl volumes, which were added to each of the sample wells. From pH 7.28 to 8.42, corresponding to NaOH concentrations from 0 to 100 mM, the absorbance differences were insignificant. At 1000 mM NaOH, the readouts scattered around the blank values, leading to a negative median of the plotted differences.

**Fig. 2:**
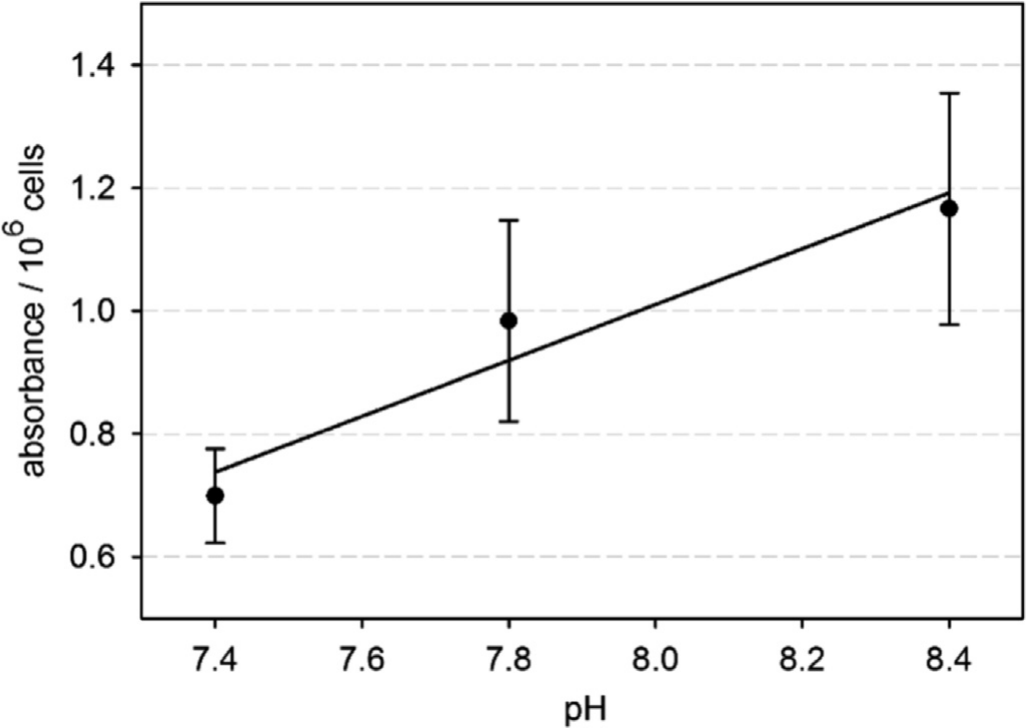
A linear regression model revealed a statistically significant (*p*<0.001) pH dependence of the measured absorbance per one million cells. A correlation coefficient of 0.79 (Pearson) proved the strong correlation between the parameters.

**Fig. 3:**
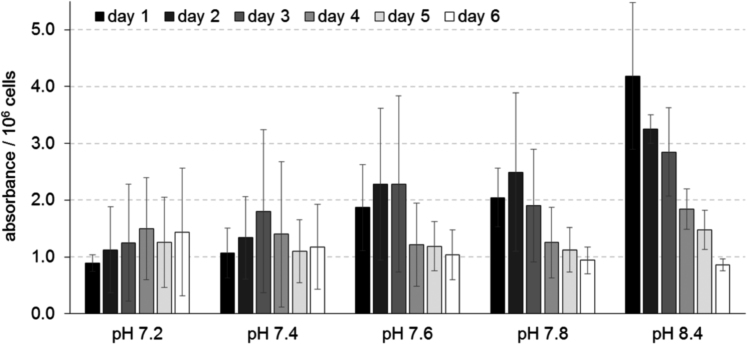
Absorbance per one million cells detected, dependent on medium pH over six days of culture. Assuming the same cellular abundance for the succinate reductase in all pH groups at the beginning of the experiment, the enzyme׳s pH-dependence should cause higher absorbance in the alkaline pH range. Indeed, the absorbance detected at pH 8.4 was greatly increased when compared with pH 7.4 on day 1 but not on day 6.

We investigated the effects of alkaline pH on the proliferation of developing osteoblasts of the osteoblast-like cell line MC3T3-E1. Cell count, cellular WST-1 metabolism, and ATP content were analyzed. The three parameters showed a pH optimum around pH 8.4 [1]. Here we present details of the tests that were carried out to verify the WST data. The first test was performed to exclude a possible pH effect on the photometric measurements (Fig. 1, and Supplementary file 1). The second test was performed to evaluate the pH-dependence of the mitochondrial succinate reductase, which metabolizes the WST-1 reagent (Fig. 2, and Supplementary file 2). The third test was conducted to analyze possible changes of the pH dependence over the culturing period of six days (Fig. 3, and Supplementary file 3).

## 2 Experimental design, materials and methods

### 2.1 Cell culture

The mouse-osteoblast precursor cell line MC3T3-E1 [2] was obtained from the German collection of microorganisms and cell culture (DSMZ, Braunschweig, Germany) and cultured as described previously [1]. For the pH experiments, carbonate-free alpha medium powder (ord. No. P03-2510, Pan Biotech, Aidenbach, Germany) was dissolved in deionized water and buffered with 20 mM HEPES (4-(2-hydroxyethyl)-1-piperazineethanesulfonic acid). The pH was adjusted to pH 7.2, 7.4, 7.6, 7.8 or 8.4.

### 2.2 General procedure for WST-assay

A standard WST proliferation assay (Roche Diagnostics GmbH, Mannheim, Germany) was used in all experiments [3]. For measurements, the medium was replaced by 500 µl of fresh medium of the respective pH supplemented with 1% WST-1 reagent. After one hour in the incubator, the optical absorbance of the supernatants was determined in a plate reader (Polarstar, BMG Labtech GmbH, Ortenberg, Germany). For this, four 100 µl samples from each measuring well were transferred to a 96-well test plate (Sarstedt AG, Nürnbrecht, Germany). The absorbance was determined at 450 nm against a reference wavelength of 620 nm. In all tests, blanks were prepared from cell-free controls with WTS-1 reagent. The blank absorbances were subtracted from those of the sample wells.

### 2.3 Test on the pH dependence of photometric measurements (Fig. 1)

Cells were seeded at high numbers (approximately 250,000 cells per well of a 12-well plate) and cultured at the standard pH of 7.4 until confluence. After incubating ten wells with WST-1 reagent at pH 7.4, twelve supernatant samples per culture well were transferred to the 96-well test plates. Subsequently, the pH in the wells of the test plate was altered by the addition of 10 µl NaOH solution taken from a concentration series of 0, 10, 100, and 1000 mM NaOH to obtain three test wells per NaOH concentration. Because the pH in the small samples could not be directly determined with our pH electrode, the same dilution ratios were prepared to determine their pH in larger volumes. For the 0, 10, 100, and 1000 mM NaOH concentrations, pH 7.28, 7.39, 8.42, and 12.72 were obtained, respectively.

### 2.4 Test on the pH dependence of mitochondrial succinate reductase (Fig. 2)

Cells were seeded at high numbers (approximately 250,000 cells per well of a 12-well plate) and cultured at the standard pH of 7.4 until confluence. The medium in the wells was replaced by 1 ml medium of either pH 7.4, 7.8 or 8.4 containing WST-1 reagent. After the assay incubation time of one hour, the samples were collected as described above. Seven repeats were done at each pH.

### 2.5 Test on the pH dependence of the WST-metabolism over time (Fig. 3)

In total, 20,000 cells were seeded per well of a 24-well plate and cultured in HEPES-buffered medium in an incubator with 95% humidity under normal atmospheric conditions for up to six days. WST-metabolism and cell count were determined daily. Based on these data, the measured absorbance per one million cells was calculated.

## Footnotes

Transparency document Transparency data associated with this article can be found in the online version at doi:10.1016/j.dib.2017.04.037.
